# Lactoferrin Disaggregates Pneumococcal Biofilms and Inhibits Acquisition of Resistance Through Its DNase Activity

**DOI:** 10.3389/fmicb.2019.02386

**Published:** 2019-10-18

**Authors:** Uriel A. Angulo-Zamudio, Jorge E. Vidal, Kamran Nazmi, Jan G. M. Bolscher, Claudia Leon-Sicairos, Brenda S. Antezana, Adrián Canizalez-Roman, Nidia León-Sicairos

**Affiliations:** ^1^CIASaP, Facultad de Medicina, Universidad Autónoma de Sinaloa, Culiacán, Mexico; ^2^Programa Regional del Noroeste para el Doctorado en Biotecnología, Facultad de Ciencias Químico-Biológicas, Universidad Autónoma de Sinaloa, Culiacán, Mexico; ^3^Hubert Department of Global Health, Rollins School of Public Health, Emory University, Atlanta, GA, United States; ^4^Department of Microbiology and Immunology, The University of Mississippi Medical Center, Jackson, MS, United States; ^5^Department of Oral Biochemistry, Academic Centre for Dentistry Amsterdam, University of Amsterdam, Amsterdam, Netherlands; ^6^Microbiology and Molecular Genetics Program, Graduate Division of Biological and Biomedical Sciences, Emory University, Atlanta, GA, United States; ^7^Unidad de Investigación, Hospital de la Mujer, Servicios de Salud de Sinaloa, Culiacán, Mexico; ^8^Departamento de Investigación del Hospital Pediátrico de Sinaloa, Servicios de Salud de Sinaloa, Culiacán, Mexico

**Keywords:** lactoferrin, disaggregates, pneumococcal biofilms, DNAse activity, resistance acquisition

## Abstract

*Streptococcus pneumoniae* colonizes the upper airways of children and the elderly. Colonization progresses to persistent carriage when *S. pneumoniae* forms biofilms, a feature required for the development of pneumococcal disease. Nasopharyngeal biofilms are structured with a matrix that includes extracellular DNA (eDNA), which is sourced from the same pneumococci and other bacteria. This eDNA also allows pneumococci to acquire new traits, including antibiotic resistance genes. In this study, we investigated the efficacy of lactoferrin (LF), at physiological concentrations found in secretions with bactericidal activity [i.e., colostrum (100 μM), tears (25 μM)], in eradicating pneumococcal biofilms from human respiratory cells. The efficacy of synthetic LF-derived peptides was also assessed. We first demonstrated that LF inhibited colonization of *S. pneumoniae* on human respiratory cells without affecting the viability of planktonic bacteria. LF-derived peptides were, however, bactericidal for planktonic pneumococci but they did not affect viability of pre-formed biofilms. In contrast, LF (40 and 80 μM) eradicated pneumococcal biofilms that had been pre-formed on abiotic surfaces (i.e., polystyrene) and on human pharyngeal cells, as investigated by viable counts and confocal microscopy. LF also eradicated biofilms formed by *S. pneumoniae* strains with resistance to multiple antibiotics. We investigated whether treatment with LF would affect the biofilm structure by analyzing eDNA. Surprisingly, in pneumococcal biofilms treated with LF, the eDNA was absent in comparison to the untreated control (∼10 μg/ml) or those treated with LF-derived peptides. EMSA assays showed that LF binds *S. pneumoniae* DNA and a time-course study of DNA decay demonstrated that the DNA is degraded when bound by LF. This LF-associated DNase activity inhibited acquisition of antibiotic resistance genes in both *in vitro* transformation assays and in a life-like bioreactor system. In conclusion, we demonstrated that LF eradicates pneumococcal-colonizing biofilms at a concentration safe for humans and identified a LF-associated DNAse activity that inhibited the acquisition of resistance.

## Introduction

*Streptococcus pneumoniae* is a Gram positive bacterium that resides in the children nasopharynx principally. These bacteria reside asymptomatically in the host, but may become invasive, resulting in pneumococcal disease (Shak et al., 2013). *S. pneumoniae* is the most common cause of community-acquired pneumonia, primarily affecting children (<5 years old) and the elderly (>65 years old). However, it can lead to a variety of other pathologies, such as otitis media, meningitis, or septicemia (Klein, 1994; Schuchat et al., 1997; Musher et al., 2000; Syrogiannopoulos et al., 2002; Regev-Yochay et al., 2004; Nunes et al., 2005). *S. pneumoniae* is a major cause of morbidity and mortality, where about 15 million individuals suffer from a pneumococcal disease and nearly 500,000 people die each year (O’brien et al., 2009). This burden of morbidity and mortality is greatly attributed to *S. pneumoniae*’s virulence factors including the ability to colonize the human host forming biofilms.

Biofilms are a community of bacteria that attach to inert or living surfaces. Once attached, the bacteria produce an exopolysaccharide matrix to encase the biofilm and protect it against the host immune response and antibiotics (Dunne, 2002). Biofilm formation initiates as the bacteria sense their own cell density via a process known as quorum sensing (QS), in which *S. pneumoniae*’s competence-stimulating peptide (CSP) plays a critical role via *comC* and the *luxS* system (Oggioni et al., 2004, 2006; Vidal et al., 2011; Yadav et al., 2018). Another major constituent of biofilms is extracellular DNA (eDNA), which can stimulate adherence, transfer genetic information, provide structural stability, and act as an energy source (Das et al., 2013).

Biofilm formation and acquisition of new traits by genetic transformation appear to be linked in *S. pneumoniae* and perhaps other naturally transformable bacteria. As such, a variety of proteins and peptides from the immune system, including lactoferrin, are natural candidates to disaggregate biofilms and/or inhibit acquisition of resistance. Lactoferrin is a cationic monomeric glycoprotein that is a part of the transferrin family because it binds iron. This protein is produced by acinary cells and glands present in different mucosal sites, at different concentrations. For example, the colostrum contains 100 μM of LF, and tears have 25 μM of LF whereas saliva, cerebrospinal fluid, and serum only contains <0.11 μM. In addition, LF is released by the secondary granules of neutrophils present in inflamed sites; its function at these sites is to sequester the iron, a crucial element for the growth and proliferation of pathogens (Vogel, 2012). LF is a multifunctional protein whose function is determined by the location in which it is found, thus being termed as a moonlighting protein (Baker and Baker, 2009).

Once ingested, LF, as other proteins, is digested in the gastrointestinal tract and this leads to the release of different peptides that also exhibit bioactive properties. Because of this evidence, peptides derived from LF have been synthetically produced. Among them, LFcin17–30 and LFampin265–284 have shown important microbicidal activities. These peptides were synthetically bound using a lysine, creating the peptide LFchimera, which is more active, compared to its peptides of origin (Van Der Kraan et al., 2004; Bolscher et al., 2009).

Due to its structure and ability to bind iron, LF presents two important effects against bacteria. LF is a bacteriostatic antimicrobial because it sequesters iron from the environment, acting as an iron chelator, inhibiting the bacterial metabolism and growth (Oram and Reiter, 1968). LF has also a demonstrated bactericidal effect mainly related to its cationic charge, which was also preserved in those LF-derivative peptides. The cationic charge allows LF to interact with negatively charged cell membrane, specifically lipopolysaccharides (LPS) in Gram negative bacteria or lipotechoic acids (LTA) in Gram positive bacteria, leading to membrane destabilization and loss of selective permeability, inducing bacterial lysis (Van Der Kraan et al., 2004, 2005; Haney et al., 2007; Leon-Sicairos et al., 2009; Lopez-Soto et al., 2009; Flores-Villasenor et al., 2010).

Bactericidal activity of LF and its peptides have been demonstrated in different bacteria such as *Escherichia coli, Staphylococcus aureus*, and *Vibrio parahaemolyticus* (Leon-Sicairos et al., 2009; Flores-Villasenor et al., 2010). LF has a high isoelectric point and can bind or interact with several molecules such as LPS, DNA, RNA, and several cell receptors. In fact, LF’s strong concentration of positive charge in residues 1–5 and in the C-terminal end of helix 1 (residues 27–30), forms the proposed binding site for DNA. In a report with *S. aureus* and LF, it was demonstrated that LF and LF peptides presented high affinity for bacterial DNA (Huo et al., 2011). Whether binding of LF to DNA leads to changes in the DNA structure, or degradation, have not been investigated. The ability of LF to bind DNA could change expression of some genes. In our previous study, we demonstrated that treatment of cultures of pneumococcus with LF decreased the transcription of the *luxS* gene, a gene whose product is involved in the regulation of early steps of biofilm formation in *S. pneumoniae* strains. Furthermore, it was also demonstrated that bLF and LF peptides decreased >90% of *S. pneumoniae* viability, perhaps through stable complexes formed among LF and LTA that were potentially liberated from the membrane, causing cell permeabilization (Leon-Sicairos et al., 2014).

The overall goal of this work was to evaluate the antimicrobial effect of LF used at concentrations spanning those found in human colostrum (100 μM) and tears (25 μM) and LF-derivative peptides against pneumococcal biofilms. The current study demonstrated a remarkable antimicrobial activity of bLF against pneumococci colonizing human respiratory cells, including antimicrobial activity against antibiotic resistant strains at a concentration safe for humans of <80 μM. We also described a non-previously recognized DNAse activity of bLF.

## Materials and Methods

### Strains and Bacterial Culture Media

*Streptococcus pneumoniae* strains utilized in this study are shown in Table 1. Strains were cultured on blood agar plates (BAP), brain heart infusion broth (BHI), or in Todd Hewitt broth containing 0.5% (w/v) yeast extract (THY). Where indicated, streptomycin (Str), trimethoprim (Tmp), or tetracycline (Tet) was added.

**TABLE 1:** *Streptococcus pneumoniae* strains used in this study.

***S. pneumoniae* strain**	**Characteristics**	**References**
D39	Serotype 2 strain	Avery et al. (1944)
TIGR4	Serotype 4, isolated from a case of bacteremia	Tettelin et al. (2001)
SPJV22	D39^str^	Lattar et al. (2018)
SPJV17	D39^tet^	Vidal et al. (2011)
SPJV27	TIGR4^Tmp^	Lattar et al. (2018)
SPJV23	TIGR4^str^	Lattar et al. (2018)
GA47281	19F^Tet, Ery, Cm^	Chancey et al. (2011b)
GA44194	19A^Tet, Ery, Cm^	Chancey et al. (2011a)

*Str, streptomycin; Tet, tetracycline; Tmp, trimethoprim; Ery, erythromycin; Cm, chloramphenicol.*

### Lactoferrin and Peptides

Bovine LF (bLF), approximately 20% iron saturated, was purchased from Abial Biotech (Spain). LPS contamination and iron concentration were previously evaluated (Cutone et al., 2014). Synthetic peptides LFcin17–30, LFampin265–284, and LFchimera were produced by solid phase peptide synthesis using Fmoc chemistry, as described previously (Bolscher et al., 2009, 2012).

### HEp-2, A549, and Detroit Cells

HEp-2 (ATCC CCL-23) human laryngeal cells, A549 (ATCC CC-L185) human lung cells, and Detroit 562 (ATCC CCL-138) human nasopharyngeal cells were used in this study, the cells were thawed and resuspended in aseptic conditions in a T-25 culture flask (Corning, NY, United States) in EMEM (Detroit and HEp-2) and F-12K (A-549) media supplemented with 10% fetal bovine serum, antibiotics 1% (Streptomycin 10 mg/ml + Penicillin 10,000 IU/ml). They were incubated at 37°C in a 5% CO_2_ atmosphere until about 85–100% confluency for future use in experiments.

### Preparation of the Inoculum for Biofilm Assays

Inoculums were prepared as previously described (Vidal et al., 2011). An overnight culture of *S. pneumoniae* grown on blood agar plates (BAP) was used to prepare a bacterial suspension in THY broth. This suspension was incubated at 37°C, 5% CO_2_ until the culture reached an OD_600_ of ∼0.2 (early log phase). Glycerol was then added to give a final 10% (v/v) and stored at −80°C until used.

### Inhibition of Initial Colonization Events by bLF and bLF Peptides

7.5 × 10^4^ CFU/ml of *S. pneumoniae* strain D39 was inoculated in 24-well plates containing A549 cells (1 × 10^4^ confluence) and treated with the following: 40 μM bLF, 10 μM LFcin17-30, LFampin265-284, or LFchimera, and 20 μM erythromycin for 4 or 6 h in F-12K medium without antibiotics and fetal bovine serum, then it was incubated at 37°C in 5% CO_2_. Supernatants were taken to determinate viability of planktonic bacteria by serial dilution and CFU/ml and the density of pneumococcal biofilm was quantified as described earlier (Vidal et al., 2013). Briefly, biofilms were washed three times with sterile 1X PBS and plates were sonicated for 15 s in a Bransonic ultrasonic water bath (Branson, Dunburry, CT, United States). This was followed by extensive pipetting to remove all attached bacteria. Detached biofilms were serially diluted and platted onto BAP, density of biofilms was expressed in CFU/ml.

### Antibacterial Activity of bLF and LF-Derivative Peptides Against Pneumococcal Biofilms

∼7 × 10^4^ CFU/ml of *S. pneumoniae* (strains with or without antibiotic resistance) were inoculated (1) onto tissue culture-treated, polystyrene, 24-well plates (Corning) and (2) HEp-2 cells (ATCC CCL-23) with a confluency of 1 × 10^4^ of cells per well that were subsequently fixed with 2% paraformaldehyde (Sigma) during 15 min. To form biofilms, samples were incubated during 4 or 8 h, respectively at 37°C in a 5% CO_2_ atmosphere. After that, planktonic cells were removed and fresh THY or cell culture medium was added. Except in the biofilm control wells, 20, 40, or 80 μM bLF, 10 μM LFamp265-285, LFcin17-30 or LFchimera were added and incubated for 6 or 12 h at 37°C with 5% CO_2_. Finally, biofilms were washed and prepared for quantification as was previously mentioned.

### Confocal Microscopy

To visualize the effect of bLF on biofilms of *S. pneumoniae*, biofilms destruction assays were done. *S. pneumoniae* TIGR4 (∼7 × 10^4^ CFU/ml) were inoculated onto Detroit 562 cells (ATCC CCL-138) grown to 85–100% confluency. To form biofilms, bacterial suspensions were incubated for 4 h at 37°C in a 5% CO_2_ atmosphere. After that, planktonic cells were removed and DMEM medium was added. Once the 4 h-biofilm was grown, 40 and 80 μM bLF was added and was incubated for 6 h at 37°C with 5% CO_2_. Following incubation, supernatants were discarded and biofilms were washed and fixed with 2% paraformaldehyde for 15 min at room temperature (RT). Biofilms were then washed two times with 1X PBS and cells were blocked with 2% BSA for 1 h at RT. For identification of the mammalian cells, sialic acid was stained with 2.5 μg/ml Wheat Germ agglutinin (WGA) conjugated with Alexa flour (Molecular probes, Invitrogen) for 15 min at RT (Wright, 1984). After that biofilms were washed once and a serotype-specific polyclonal antibody (labeled with Alexa 488, Molecular Probes, Thermo Fisher Scientific, Grand Island, NY, United States) was used to stain *S. pneumoniae* (40 μg/ml) for 1 h at RT (Wu et al., 2017). Then biofilms were washed once and mounted with ProLong Diamond antifade mountant with DAPI (Molecular Probes, Thermo Fisher Scientific, Grand Island, NY, United States). Confocal images were obtained using an Olympus FV1000 confocal microscope and were analyzed with the software ImageJ version 1.49k.

### Purification and Quantification of Extracellular DNA

*Streptococcus pneumoniae* (∼7 × 10^4^ CFU/ml) was inoculated onto (1) tissue culture-treated polystyrene 24-well plates (Corning, NY, United States) or (2) onto 1 × 10^4^ HEp-2 cells (ATCC CCL-23) that had been fixed with 2% paraformaldehyde (Sigma Aldrich, St. Luis, MO, United States) and extensively washed to remove the PFA. Inoculated plates, or infected cells, were incubated during 4 h or 8 h, at 37°C with 5% CO_2_ to form biofilms. Treatment with bLF (40 and 80 μM) or bLFpeptides (10 μM) was subsequently added as was indicated before. The supernatants were then collected and centrifuged for 10 min at 12,000 × *g* in a refrigerated centrifuge (Eppendorf, Hamburg, Germany) and filtered by using a 0.2 μM syringe filter. They were subsequently mixed with a 0.5 volume of ethanol and vortexed for 10 s. The DNA from the supernatants was then purified by using the QIAamp DNA minikit (QIAGEN, Hilden, Germany) following the manufacturer’s instructions. To quantify amounts of extracellular DNA, quantitative PCR (qPCR) assay, targeting the *lytA* gene was utilized (Carvalho et al., 2007). Reactions were performed with IQ^TM^ SYBR green super mix (BioRad, Hercules, CA, United States), 300 nM of each primer and 4 ng/μl of DNA template. Reactions were run in duplicate using a CFX96 Real-Time PCR Detection System (Bio-Rad, Hercules, CA, United States) with the following conditions: 1 cycle at 55°C for 3 min, 1 cycle at 95°C for 2 min, 40 cycles of 95°C for 15 s, 55°C for 1 min, and 72°C for 1 min. Melting curves were generated utilizing a cycle of 95°C for 1 min, 65°C for 1 min, and 80 cycles starting at 65°C with 0.5°C increments. For quantification purposes, standards containing 1 × 10^3^, 1 × 10^2^, 1 × 10^1^, 1 × 10^0^, 1 × 10^–1^, 5 × 10^–2^, or 1 × 10^–3^ pg of *S. pneumoniae* DNA were run in parallel to generate a standard curve and amounts of eDNA were calculated by using the software Bio-Rad CFX manager (Hercules, CA, United States).

#### EMSA Assay

*Streptococcus pneumoniae* D39 (∼7 × 10^4^ CFU/ml) was inoculated into THY medium and incubated for 2.5 h at 37°C with 5% CO_2_. The suspension was centrifuged for 10 min at 12,000 × *g* in a refrigerated centrifuged. DNA was purified by using the Wizard Genomic DNA Purification Kit (PROMEGA, Madison, WI, United States) following the manufacturer’s instructions. To analyze the interactions between bLF and DNA, both bLF and albumin proteins were used and evaluated with the Electrophoretic Mobility-Shift Assay (EMSA) kit (Thermo Fisher Sicientific, Waltham, MA, United States) following the manufacturer’s instructions. To visualize the DNA-protein interaction, the samples were separated in a 2% agarose gel and dyed with gel red (Invitrogen, CA, United States). Gels were imaged using the Kodak model E1 logia 100 imaging system.

### DNA Decay Rate

DNA purified as above was incubated with 40 μM bLF or 40 μM albumin for 80 min at 37°C. To measure DNA decay, DNA concentrations were monitored every 10 min using the Nanodrop 2000 spectrophotometer (Thermo Scientific, Waltham, MA, United States).

### Transformation Frequency Assay

To evaluate the effect of bLF on genetic transformation of *S. pneumoniae*, we performed the classic transformation assay. The experiment was carried out in *S. pneumoniae* D39 competent cells using standard procedures. To induce transformation and test the effect of bLF on *S. pneumoniae*’s ability to transform, a positive control consisting of competent cells, 100 ng of competence-stimulating peptide 1 (CSP1 [EMRLSKFFRDFILQRKK]), 100 ng/mL of DNA purified from strain SPJV22 encoding resistance to Str (200 μg/ml), in complete transformation media was mixed. To test bLF activity against the transformation process, 40 and 80 μM bLF were added to the same reaction as that of the positive control. The negative control lacked CSP1 to prevent transformation. Every sample had a final volume of 200 μl (Havarstein et al., 1995). Samples were incubated for 2 h at 37°C and subsequently plated in the presence or absence of streptomycin. The plates were incubated at 37°C with 5% CO_2_ overnight and counted the next day. Transformation frequency was calculated by dividing the number of streptomycin-resistant transformants by the total population obtained on blood agar plates without streptomycin.

### Effect of bLF on Recombination Frequency Between Two Strains of *S. pneumoniae* in a Bioreactor Model

To evaluate the effect of bLF on recombination frequency (rF), we followed the methodology established by Lattar et al. (2018) with a small modification. Detroit 562 cells (ATCC CCL-138) were grown on snapwell filters (Corning, Corning, NY, United States) for about 4–5 days (until polarized). Snapwells with cells were transferred to the bioreactor chambers and the cells were washed with EMEM media without antibiotics supplemented with 5% fetal serum bovine at a flow rate of 0.20 ml/min using a Master Flex L/S precision pump system (Cole-Parmer, Vernon Hills, IL, United States). Bioreactor chambers were then inoculated with ∼1 × 10^6^ CFU/ml of *S. pneumoniae* D39^tet^ and TIGR4^str^. One chamber was treated with 40 μM bLF, a second with 80 μM bLF, and a third served as a negative control with no treatment. Each chamber was incubated at ∼35°C. After 8 h of incubation in the bioreactor, biofilms were harvested by sonication for 15 s in a Bransonic ultrasonic water bath. This was followed by extensive pipetting to remove all attached bacteria. To obtain the bacterial density, serial dilutions were done and samples were plated on Tet (1 μg/ml) + Str (200 μg/ml) BAP. These plates were incubated at 37°C with 5% CO_2_ overnight. To calculate rF, bacteria growth in obtained from BAP with str and tet was divided between bacteria growth in Tet. Recombination frequency was calculated by dividing the number of Tet + Str-resistant recombinants by the total population obtained on blood agar plates without antibiotics.

### Statistical Analyses

All experiments were performed in triplicate and analyzed by ANOVA test and student *t*-test using the SigmaPlot software version 12.0 (CA, United States).

## Results

### Lactoferrin and LF-Derivative Peptides Inhibit *S. pneumoniae* Colonization of Human Respiratory Cells

**FIGURE 1:**
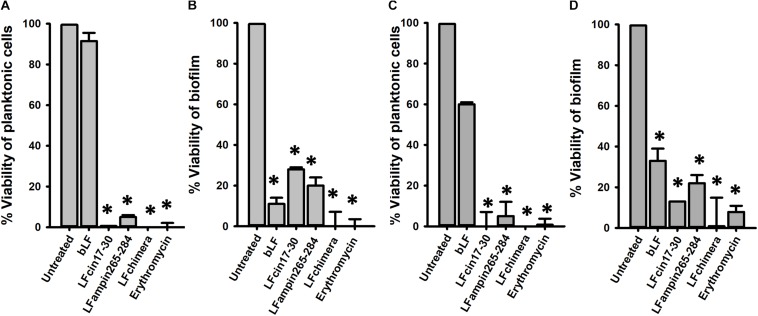
Bovine Lactoferrin and LF-derived peptides inhibit the viability and attachment of pneumococci present in lung cells and supernatants. Human lung cells A549 were inoculated with 7.5 × 10^4^ CFU/ml *Streptococcus pneumoniae* strain D39 and simultaneously treated with 40 μM bLF, 10 μM LFcin17-30, 10 μM LFampinn265-284, 10 μM LFchimera, and 20 μM Erythromycin. Infected cells were incubated for 4 h **(A,B)** or 6 h **(C,D)** at 37°C with 5% CO_2_. Counts of planktonic pneumococci were obtained in **(A,C)** while biofilm pneumococci were harvested and counted **(B,D)**. Asterisks indicate statistical significance calculated using the ANOVA *t* test (*p* < 0.05). Viable counts of pneumococci in the untreated control were adjusted to 100% and viability on those treated was calculated.

We first sought to evaluate if bLF and LF-derived peptides would inhibit pneumococcal colonization on human respiratory cells, the first step of pneumococcal biofilm formation. To assess this, confluent cultures of human A549 cells were inoculated with *S. pneumoniae* strain D39 and treated with bLF, or LF-derived peptides. As a control, some A549-infected cells were treated with erythromycin. Infected and treated human lung cells were incubated for 4 or 6 h after which the viability of planktonic cells or colonizing biofilms was evaluated by dilution and platting. Experiments demonstrated that the density on planktonic pneumococci was not affected by the treatment with bLF while LF-derivative peptides, and erythromycin, eradicated planktonic bacteria (Figure 1A).

Despite bLF did not affect planktonic pneumococci, the viability of pneumococci colonizing human A549 cells and that had been treated with bLF, decreased >90% (Figure 1B). As expected because of the bactericidal activity against planktonic bacteria, LFcin17–30 and LFampin265–284 decreased ∼70 and ∼80%, respectively, the viability of pneumococci colonizing lung cells while LFchimera eradicated colonizing pneumococci at the same extent as the antibiotic erythromycin did (Figure 1B). A similar antibiotic effect was observed when infected human lung cells were treated with bF, and LF-derivative peptides, and incubated for 6 h (Figures 1C,D).

### Lactoferrin Eradicate *S. pneumoniae* Biofilms

**FIGURE 2:**
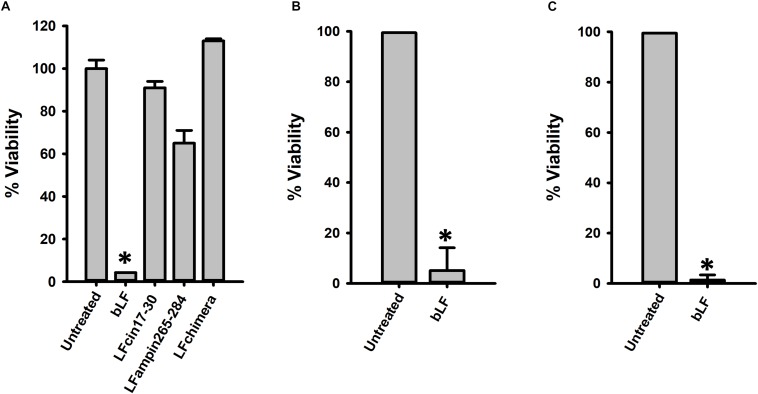
Lactoferrin but not its derived peptides destroy preformed *Streptococcus pneumoniae* biofilms. 7.5 × 10^4^ CFU/ml *S. pneumoniae* strain D39 were inoculated in a 24-well plate containing THY and then plates were incubated for 4 h **(A,B)** or 8 h **(C)** at 37°C with 5% CO_2_. After that, planktonic cells were removed, biofilms were washed and then treated with 40 μM bLF, 10 μM LFcin17-30, 10 μM LFampinn265-284, and 10 μM LFchimera for 6 h **(A,C)** or 12 h **(B)**. Treated biofilms were harvested, diluted, and platted on blood agar plates to obtain viable counts. Experiments were repeated at least three times. Standard deviation of the mean is shown. Asterisks indicate statistical significance calculated using the ANOVA test (*p* < 0.05). Viable counts of pneumococci in the untreated control were adjusted to 100% and viability on those treated was calculated.

Having demonstrated activity against planktonic and colonizing pneumococci, we then assessed anti-biofilm activity of bLF and LF-derived peptides against pneumococcal biofilms. To assess this, biofilms made by *S. pneumoniae* strain D39 for 4 or 6 h, were challenged with 40 μM bLF, or with 10 μM of peptides LFcin17–30, LFamp264–285, and LFchimera. Biofilms were treated for 6 or 12 h. Unexpectedly, treatment of 4 h preformed biofilms with LF-derivative peptides did not induce a significant reduction of pneumococcal biofilms (Figure 2A). In contrast, eradication of biofilms was observed in those pneumococcal biofilms treated with bLF, whether biofilms had been preformed during 4 or 6 h, and at both treatment times, 6 or 12 h (Figures 2B,C).

**FIGURE 3:**
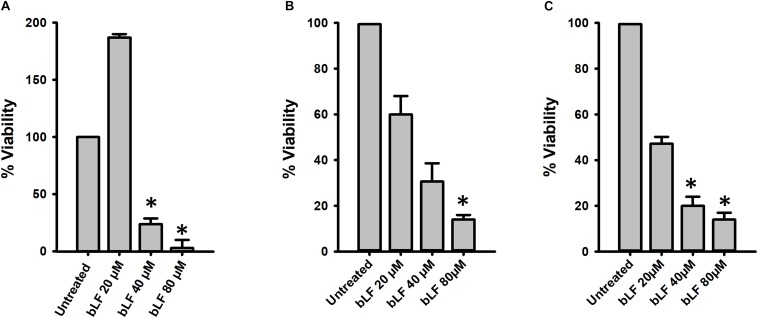
Bovine Lactoferrin eradicates antibiotic-resistance strains of *Streptococcus pneumoniae* biofilms. *S. pneumoniae* strains TIGR4^Tmp^ **(A)**, GA47281^Tet+Ery+Cm^ **(B)**, and GA44194^Tet+Ery+Cm^ **(C)**, were inoculated in a 24-well plate containing THY. The plate was incubated for 4 h at 37°C with a 5% CO_2_ atmosphere. Planktonic cells were removed and the remaining biofilms were washed once and bovine Lactoferrin (bLF) was added at the indicated concentration and incubated for 6 h. Treated biofilms were harvested, diluted, and platted on blood agar plates to obtain biofilm viable counts. Experiments were repeated at least three times. Standard deviation of the mean is shown. Asterisks indicate statistical significance calculated using the ANOVA test (*p* < 0.05). The viability (%) refers to number of live cells present in samples relative to untreated cells.

We additionally tested the antibacterial effect of bLF against pneumococcal biofilms of antibiotic resistant strains. Biofilms of strain TIGR4^Tmp^ that we engineered to bear trimethoprim resistance or two naturally multidrug resistant strains, GA47281^Tet+Ery+Cm^ and GA44194^Tet+Ery+Cm^ were challenged during 6 h with bLF. Figure 3 results demonstrated a dose-dependent decrease of the viability of bLF-treated biofilms, in comparison with untreated bacteria.

**FIGURE 4:**
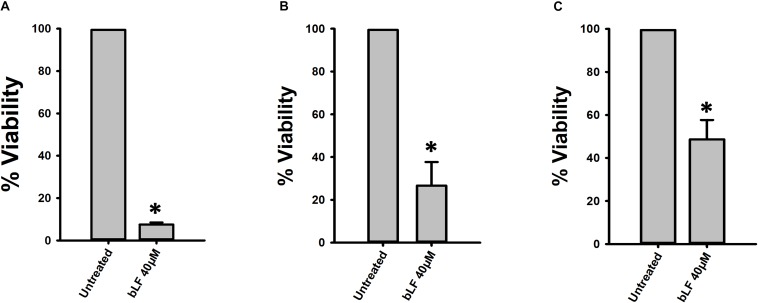
Lactoferrin eradicates pneumococcal colonization on human HEp-2 cells. *Streptococcus pneumoniae* strain D39 (7.5 × 10^4^ CFU/ml) was inoculated in cultures of human HEp-2 cells and incubated for 4 h **(A,B)** or 8 h **(C)** at 37°C with 5% CO_2_. Planktonic cells were removed and biofilms were washed and treated with bovine Lactoferrin (bLF) for 6 h **(A,C)** or 12 h **(B)**. Treated pneumococci were harvested, diluted, and platted on blood agar plates to obtain viable cells. Experiments were repeated at least three times. Standard deviation of the mean is shown. Asterisks indicate statistical significance calculated using the ANOVA test (*p* < 0.05). Viable counts of pneumococci in the untreated control were adjusted to 100% and viability on those treated was calculated.

**FIGURE 5:**
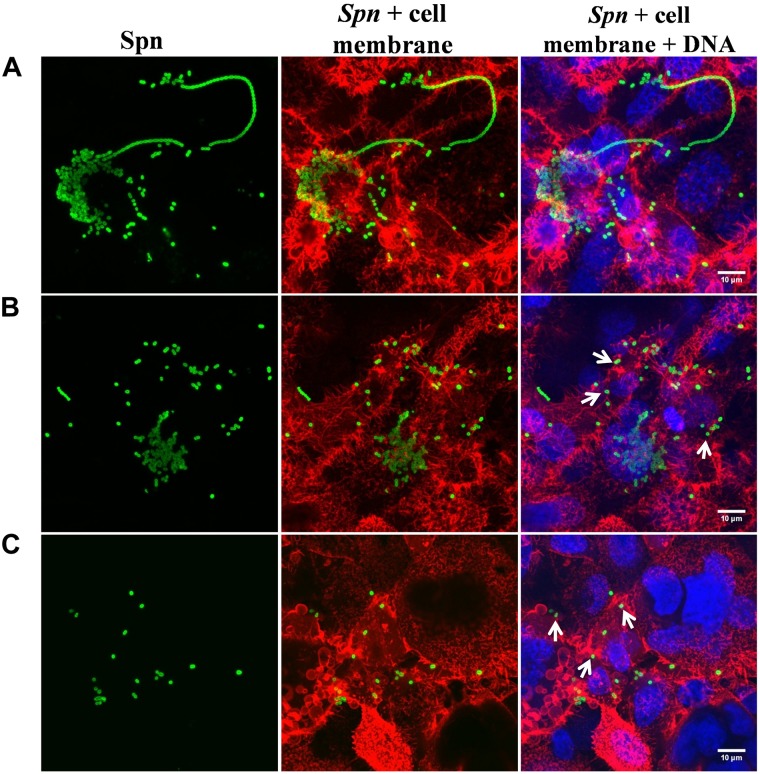
Lactoferrin eradicates preformed *Streptococcus pneumoniae* biofilms in nasopharynx cells. *S. pneumoniae* (spn) strain TIGR4 (7.5 × 10^4^ CFU/ml) was inoculated in a 24-well plate containing Detroit cells and plates were incubated for 4 h at 37°C with 5% CO_2_. Planktonic cells were removed and biofilms were washed and then untreated **(A)** or treated with bovine Lactoferrin bLF, 40 and 80 μM (**B,C**, respectively) for 6 h. Treated biofilms were slowly washed once with PBS, fixed with 2% *P*-formaldehyde, and stained with Wheat germ agglutinin (WGA) for the cell membrane, antibody serotype-specific polyclonal for *S. pneumoniae* and DAPI for DNA. Confocal images with close up of 10 μM were obtained using an Olympus FV1000 confocal microscope and were analyzed with ImageJ version 1.49k. Arrows indicate the decrement of pneumococcal cells in biofilms.

To further investigate a potential therapeutic and/or prophylactic use, we next determined the effect of bLF on pneumococcal biofilms that had been preformed on human pharyngeal cells. Similarly to the above experiments, bLF induced a statistically significant reduction of viability of pneumococcal biofilms that had been preformed on human pharyngeal cells (Figure 4). Treatment for 6 and 8 h induced a ∼80% reduction of viable pneumococci (Figures 4A,B), while 12 h post-treatment a decreased viability of only ∼60% was observed indicating that bLF was losing its activity and therefore pneumococci that remained viable after 8 h of incubation started to growth (Figure 4C). Pharyngeal cells colonized by pneumococci, and those colonized and then treated with bLF, were visualized by confocal microscopy. In line with experiments where viable counts were the readout, a number of pneumococci colonizing pharyngeal cells, and with the hallmark pneumococcal infection forming bacterial chains, were observed in the micrographs of the untreated control (Figure 5). These chains were absent in pharyngeal cells infected with pneumococci and treated with bLF at both concentration used, 40 and 80 μM. Moreover, a reduction in the number of pneumococcal cells was observed in cells treated with 40 μM while in those treated with 80 μM pneumococci were scarce (Figure 5, bottom panels). Altogether, our experiments demonstrated a strong anti-bacterial activity of bLF against pneumococci already colonizing human pharyngeal cells.

### Lactoferrin Degrades eDNA Release During Biofilm Formation

**FIGURE 6:**
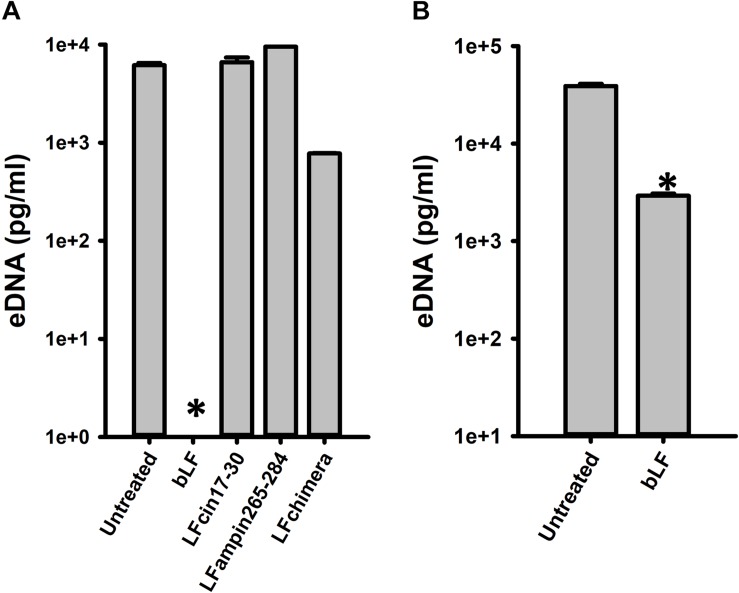
Bovine lactoferrin digest eDNA in preformed *Streptococcus pneumoniae* biofilms. *S. pneumoniae* strain D39 (7.5 × 10^4^ CFU/ml) was inoculated in a 24-well plate containing THY and incubated for 4 h **(A)** or 8 h **(B)** at 37°C with a 5% CO_2_ atmosphere. Planktonic cells were removed; biofilms washed once and then treated with 40 μM bLF, 10 μM, LFcin17-30, 10 μM LFampinn265-284, and 10 μM LFchimera for 6 h **(A)** or 12 h **(B)**. After that, the supernatants were removed and DNA extracted. eDNA quantification was performed by using qPCR. Experiments were repeated at least three times. Standard deviation of the mean is shown. Asterisks indicate statistical significance calculated using the ANOVA test (*p* < 0.05).

To begin evaluating the molecular mechanism utilized by bLF to eradicate pneumococcal biofilms, we assessed the effect of bLF on a main component of the biofilm matrix, the extracellular eDNA. Fascinatingly, eDNA in pneumococcal biofilms formed for 4 h was completely absent in biofilms treated with bLF compared to the untreated control that contained 10 μg/ml of eDNA, or compared to those treated with LF-derivate peptides in which eDNA was intact (Figure 6A). eDNA was also significantly reduced in biofilms that had been preformed for 8 h and then treated with bLF for 6 h (Figure 6B).

**FIGURE 7:**
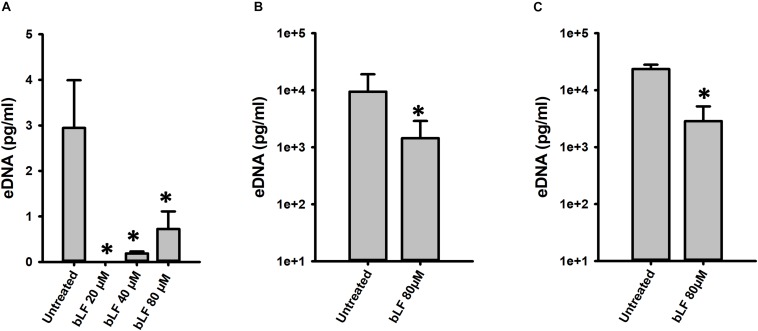
Bovine lactoferrin degrades eDNA from antibiotic-resistance strains of *Streptococcus pneumoniae. S. pneumoniae* strains TIGR4^Tmp^ **(A)**, GA47281^Tet+Ery+Cm^ **(B)**, and GA44194^Tet+Ery+Cm^ **(C)** were inoculated in a 24-well plate containing THY. Then, the plate was incubated for 4 h at 37°C with a 5% CO_2_ atmosphere. Planktonic cells were removed, biofilms washed once, and bovine Lactoferrin (bLF) was added at the indicated concentrations and incubated for 6 h. The supernatants were removed, filter-sterilized to eliminate bacteria in supernatant and DNA was extracted. eDNA quantification was performed by using qPCR. Experiments were repeated at least three times. Standard deviation of the mean is shown. Asterisks indicate statistical significance calculated using the ANOVA test (*p* < 0.05).

Degradation of eDNA was also evaluated in biofilms formed by antibiotic resistance strains (TIGR4^Tmp^, GA47281^Tet+Ery+Cm^, and GA44194^Tet+Ery+Cm^) challenged with 20, 40, and 80 μM bLF. In all experiments, a statistically significant reduction of eDNA was observed in biofilms treated with bLF (Figure 7). Whereas eDNA from reference strains D39 and TIGR4 was completely absent (i.e., 1 × 10^3^-fold reduced) after the treatment with 40 μM of bLF, eDNA of naturally resistant pneumococcal strains was 10-fold reduced, but not completely degraded, indicating that those strains might have evolved a mechanism to partially inactivate the DNAse activity of bLF.

**FIGURE 8:**
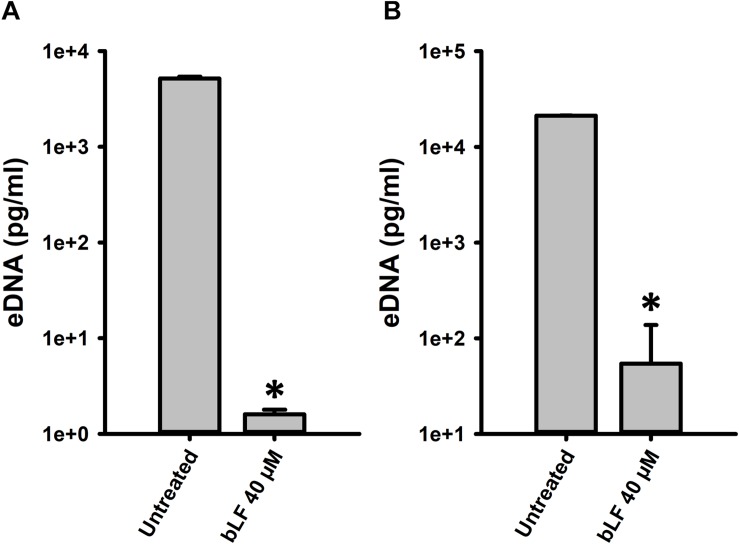
Bovine lactoferrin degrades eDNA in preformed *Streptococcus pneumoniae* biofilms in Hep-2 cells. *S. pneumoniae* strain D39 (7.5 × 10^4^ CFU/ml) was inoculated in a 24-well plate containing Hep-2 cells and incubated for 4 h **(A,B)** at 37°C with a 5% CO_2_ atmosphere. Then, planktonic cells were removed, biofilms washed once, then treated with bovine Lactoferrin (bLF) for 6 h **(A)** or 12 h **(B)**. After that, the supernatants were removed and DNA extracted. eDNA quantification was performed by using qPCR. Experiments were repeated at least three times. Standard deviation of the mean is shown. Asterisks indicate statistical significance calculated using the ANOVA test (*p* < 0.05).

We finally evaluated whether degradation of eDNA by bLF would be observed in the context of colonization of human pharyngeal cells. Accordingly, eDNA from strain D39 colonizing human cells was ∼10,000-fold reduced in the supernatants of cells infected with pneumococci and treated with bLF for 6 or 12 h in comparison to those infected cells that were left untreated (Figure 8). Overall our data indicate that bLF bears DNAse activity.

### Lactoferrin Binds and Degrades DNA Inhibiting Acquisition of Resistance by Pneumococcal Strains

**FIGURE 9:**
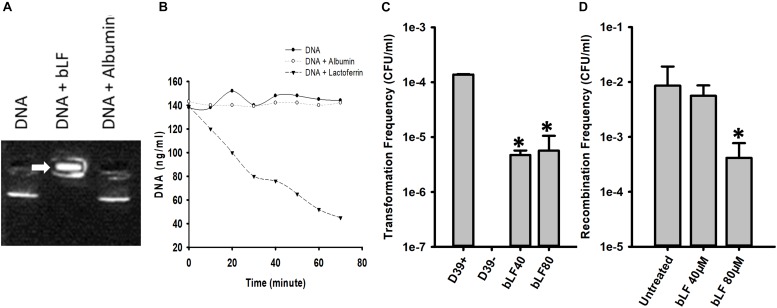
Bovine lactoferrin binds and degrades DNA, decreasing transformation and recombination frequencies of *Streptococcus pneumoniae. S. pneumoniae* D39 DNA was mixed with bovine Lactoferrin or albumin (40 μM) for 15 min. An electrophoretic mobility shift assay was done and was separated with 2% agarose gel **(A)**. The mix of DNA-LF or DNA-albumin were also measured every 10 min using nanodrop **(B)**. Competent *S. pneumoniae* D39 was mixed with DNA and CSP 1 in the presence or absence of bLF and incubated at 37°C for 2 h. Transformation frequencies were calculated **(C)**. Bioreactor chambers with D39^tet^ and TIGR4^str^ were co-incubated for 8 h in the presence of bLF (40 and 80 μM) or were left untreated. Bacteria were then counted and the recombination frequencies were calculated **(D)**. Standard deviation of the mean is shown. Asterisks indicate statistical significance calculated using the ANOVA test (*p* < 0.05).

If bLF degrades DNA then it should first bind it; we therefore performed an EMSA assay to investigate this possibility with DNA purified from cultures of strain D39. Figure 9A demonstrated that bLF bound genomic DNA, trapping the DNA into the well whereas untreated pneumococcal DNA, or pneumococcal DNA that had been incubated with another serum protein albumin, migrated into the gel. To further investigate degradation of genomic DNA by bLF, we conducted a time-course study of DNA decoy. Genomic DNA (∼140 ng/ml) was left untreated, or treated with bLF, or albumin for up to 60 min. Statistically significant degradation of genomic DNA was observed soon after 20 min of incubation with bLF and continued decreasing through the incubation time (Figure 9B). In contrast, DNA signal remained intact in untreated DNAs or in those DNA samples treated with albumin (Figure 9B). Together these experiments demonstrate that bLF binds DNA to induce its degradation.

We then hypothesize that if bLF degrades DNA, it should inhibit acquisition of resistance. We first tested whether acquisition of resistance in classic transformation reactions would be inhibited by bLF. As shown in Figure 9C, in transformation reactions containing competent D39 pneumococci, DNA encoding resistance to streptomycin, and bLF, the transformation frequency (tF) was significantly decreased in comparison to the tF obtained in the untreated control.

Given that acquisition of resistance by pneumococcal strains is stimulated when strains D39 and TIGR4 are inoculated in pharyngeal cells and incubated under the dynamic system of a bioreactor, we assessed whether treatment with bLF would inhibit acquisition of resistance by evaluating the recombination frequency (rF) in this life-like environment. In bioreactor chambers containing human pharyngeal cells and inoculated with strains D39^Tet^ and TIGR4^Str^, a rF of ∼1 × 10^–2^ of pneumococci bearing tetracycline and streptomycin resistance was obtained (Figure 9D). Bioreactors chambers incubated also with 40 μM of bLF yielded a similar rF to that in untreated bioreactor chambers, whereas in chambers incubated with 80 mM of bLF the rF was 100-fold reduced confirming that bLF inhibit the acquisition of resistance by degrading eDNA.

## Discussion

In the present report, we utilized *in vitro* models to mimic two important biological events leading to persistence of pneumococcus in the human airways (i.e., initial colonization events and persistent pneumococcal biofilms), to evaluate the effect of physiologically relevant concentrations of LF and LF-derivative peptides on biofilm persistence. We first demonstrated that bLF, and LF-derivative peptides, inhibited initial colonization events on human respiratory cells. The mechanism(s) that blocked colonization was different for bLF compared to that of LF-derivative peptides. For the latter, inhibition of colonization was caused by the antibacterial activity of LF-derivative peptides against planktonic pneumococci. The mechanism utilized by bLF to inhibit pneumococcal colonization was different since bLF presented low antibacterial activity against planktonic bacteria. In line with our experiments, an effect for LF against colonization by other organisms such as *Pseudomonas aeruginosa*, *Streptococcus mutans*, and *Candida albicans* has been demonstrated (Berlutti et al., 2004; O’may et al., 2009; Morici et al., 2016). The iron-chelating activity of LF was demonstrated as the main factor (Singh et al., 2002). The current study, and a previous publication by our laboratories (Leon-Sicairos et al., 2014), allow us to speculate that bLF inhibits biofilm formation through two different mechanisms: (1) a decreased of *luxS* gene expression, a gene that regulates biofilm formation (Vidal et al., 2011), and the binding of LF to pneumococcal surface protein A (PspA) thus inhibiting adherence of the pneumococcus to human epithelial cells. Blocking adherence through PspA binding has been linked to an increased number of non-adherent pneumococci bacteria (Shaper et al., 2004), such as it was demonstrated in experiments presented in Figures 1B,D.

Despite having a strong bactericidal effect against planktonic bacteria inoculated onto human respiratory cells, to our surprise, LF-derivative peptides did not have an effect against pneumococci already forming a biofilm. Given that LF-derivative peptides were very efficient for eradicating planktonic pneumococci, these peptides likely target metabolically active bacteria and not pneumococci in quiescent state forming a biofilm. The possibility exist that an increased density of pneumococci in the biofilms state, versus planktonic bacteria challenged with LF-peptides, was a factor to the inability of LF-derivative peptides to affect pneumococcal biofilms.

The current study, however, demonstrated that bLF eradicates pneumococci forming biofilms at two different density of biofilm bacteria, those already formed after 4 h of incubation and 8 h post-inoculation. Moreover, the density of these pneumococcal biofilms decreased whether biofilms were formed on abiotic substrates or on human pharyngeal cells, which bears both prophylactic and therapeutic potential to combat nasopharyngeal colonization or pneumococcal disease such as otitis media or sinusitis. The concentration of LF that eradicated pneumococcal biofilms (20, 40, and 80 μM) was similar to the concentrator of LF found in colostrum (100 μM) and tears (25 μM) and therefore it is safe for humans. Both colostrum and tears protect the human host from bacterial infections, in part because of the antimicrobial activity of LF.

It has been reported that LF has anti-biofilm properties when mixed with other molecules. For example, Ammons and colleagues (2009) demonstrated that LF penetrates *P. aeruginosa* biofilms and reduced 1 log reduction in viability, but in their experiments a combination LF and xylitol reduced ∼3 unit/logarithmic (Ammons et al., 2009). Sheffield et al. (2012) tested LF to disrupt *E. coli* biofilms; LF reduced more than 90% of *E. coli* biofilms and 100% of *Klebsiella pneumoniae* biofilms (Sheffield et al., 2012) whereas an anti-biofilm effect against the Gram positive *S. mutans* decreasing the density of biofilms has also been demonstrated (Allison et al., 2015). These studies, however, were conducted using abiotic substrates to produce biofilms of Gram negative bacteria. Our current studies with the Gram positive *S. pneumoniae* are in line with these mentioned above but we additionally demonstrated that bLF eradicates biofilms made on biologically relevant substrates (i.e., human respiratory cells) and therefore supports a potential therapeutic and/or prophylactic application for LF.

How bLF induced the decrease of the density of *S. pneumoniae* biofilms? There are a number of possibilities. For example, given that LF chelates iron and although the metabolism in biofilms decreases these quiescent bacteria still may require iron to maintain its structure and/or vital activities. Another possibility is its capacity to interact with negative components of bacteria such as LPS (Ellison et al., 1988; Leon-Sicairos et al., 2014). Besides these two possibilities, we experimentally provided with a mechanism when an important component of the pneumococcal biofilm matrix was evaluated, eDNA and found a significantly decrease of eDNA in the supernatant. Since eDNA is an anionic molecule, our mechanistic experiments demonstrated that in fact bLF binds DNA and that this biding precedes DNA degradation. As expected, degradation was observed in different pneumococcal strains, including multi-drug resistant pneumococci, although at different extents indicating that other individual, strain specific, components of the biofilm matrix interfered with bLF-induced degradation of eDNA. All evidence together led us to formulate the following hypothesis that bLF decreased the density of pneumococcal biofilms by chelating iron necessary to maintain biofilm viability, and binding to negatively charged components of the biofilm matrix such as eDNA, thus degrading this important component of the matrix. Significant more efforts will be required to fully dissect the mechanism of biofilm eradication.

It very interesting that concentration that bLF that we used to demonstrate the antimicrobial activity against pneumococcal biofilms is inside of range or lower of some physiological fluids of human. The higher concentration that we used was 80 μM, lower that in colostrum 100 μM (Sánchez et al., 1992). It means that concentration used in this work couldn’t present adverse effect in human, in fact 40 μM of LF was sufficient to alter the biofilms structure, concentration that is inside of milk range (20–60 μM) (Ford et al., 1977; Zavaleta et al., 1995; Hamosh, 1998). The low concentration of LF in upper airways of human (6.25–12.5 μM) could to influence to allow the colonization of pneumococcus (Dubin et al., 2004).

Our studies identified an activity of bLF that had not previously been recognized, DNAse activity (Bennett et al., 1986). The aminoacid threonine play a central role in DNA hydrolysis in active site of DNAse 1, this aromatic residue induces distortion of DNA (Parsiegla et al., 2012), in comparison with the active zone of LF to bind DNA located in C-terminal in end of helix in the aminoacids residues 27–30, which contains threonine, fact that could help in DNAse activity of bLF (Baker and Baker, 2009). DNase has been successful used to disaggregates pneumococcal biofilms (Hall-Stoodley et al., 2008), and, of course, DNase has a profound effect on pneumococcal acquisition of new traits (i.e., capsule genes, antibiotic resistance genes) by recombination via transformation. Treatment with DNase completely blocked recombination via transformation in a life-like system such as that utilized in the current study (Lattar et al., 2018). Accordingly, our studies demonstrated that treatment with bLF rendered the pneumococcus unable to uptake DNA whether the transformation reaction was performed *in vitro* or using our model of recombination in a life-like bioreactor system. Therefore, bLF not only has the capacity to eradicate colonizing pneumococcal biofilms, but also it appears to prevent recombination by its DNase activity. Because human lactoferrin (hLF) has ∼72% aminoacids homology with bLF (calculated by BLAST analysis), it is likely that hLF is also able to degrade DNA. We in the field have speculated for a number of years now that the future of therapeutics would include targeting important components of the pneumococcal transformation machinery including the competence stimulating peptide (CSP) and eDNA (Hakenbeck, 2000; Moreno-Gamez et al., 2017; Salvadori et al., 2019). Whereas this was an interesting recommendation, it has not been successfully implemented as of yet, perhaps due to the lack of specific molecules that are safe for humans such as lactoferrin.

## Data Availability Statement

All datasets generated for this study are included in the manuscript/supplementary files.

## Author Contributions

UA-Z, JV, and NL-S conceived and designed the study. UA-Z, KN, JB, CL-S, and BA collected the data. UA-Z, AC-R, JV, and NL-S analyzed and interpreted the data. UA-Z and BA drafted the manuscript. AC-R, JV, and NL-S critically revised the manuscript. UA-Z, AC-R, KN, JB, CL-S, BA, JV, and NL-S approved the final version of the manuscript.

## Conflict of Interest

The authors declare that the research was conducted in the absence of any commercial or financial relationships that could be construed as a potential conflict of interest.
